# EBs Recognize a Nucleotide-Dependent Structural Cap at Growing Microtubule Ends

**DOI:** 10.1016/j.cell.2012.02.049

**Published:** 2012-04-13

**Authors:** Sebastian P. Maurer, Franck J. Fourniol, Gergő Bohner, Carolyn A. Moores, Thomas Surrey

**Affiliations:** 1 Cancer Research UK London Research Institute, Lincoln's Inn Fields Laboratories, 44 Lincoln's Inn Fields, London WC2A 3LY, UK; 2 Institute of Structural and Molecular Biology, Birkbeck College, London WC1E 7HX, UK; 3 European Molecular Biology Laboratory, Cell Biology and Biophysics Unit, Meyerhofstrasse 1, 69117 Heidelberg, Germany

## Abstract

Growing microtubule ends serve as transient binding platforms for essential proteins that regulate microtubule dynamics and their interactions with cellular substructures. End-binding proteins (EBs) autonomously recognize an extended region at growing microtubule ends with unknown structural characteristics and then recruit other factors to the dynamic end structure. Using cryo-electron microscopy, subnanometer single-particle reconstruction, and fluorescence imaging, we present a pseudoatomic model of how the calponin homology (CH) domain of the fission yeast EB Mal3 binds to the end regions of growing microtubules. The Mal3 CH domain bridges protofilaments except at the microtubule seam. By binding close to the exchangeable GTP-binding site, the CH domain is ideally positioned to sense the microtubule's nucleotide state. The same microtubule-end region is also a stabilizing structural cap protecting the microtubule from depolymerization. This insight supports a common structural link between two important biological phenomena, microtubule dynamic instability and end tracking.

## Graphical abstract

**Graphical Abstract:**

**Figure:**
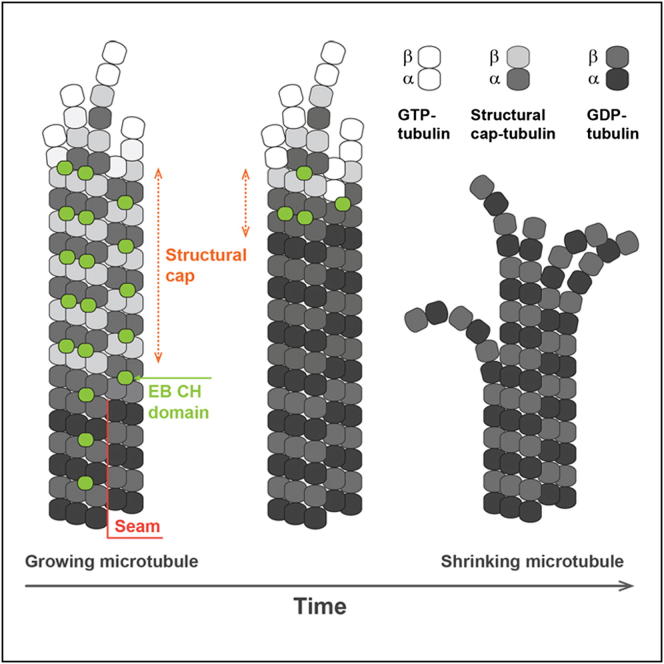

**Highlights:**

► End-binding protein (EB) Mal3 bridges microtubule protofilaments except at the seam ► Mal3 binds next to the tubulin GTPase site and is positioned to sense GTP hydrolysis ► EBs recognize a stabilizing structural cap at growing microtubule ends ► EB end tracking and dynamic instability are mechanistically linked

## Teaser

Subnanometer reconstruction of a microtubule end-binding protein (EB) in complex with microtubules reveals interactions with the GTP cap that protects the growing end from depolymerization. The results suggest a mechanism for how EBs track dynamic changes in filament length and provide a moving platform for recruiting regulatory proteins.

## Introduction

The microtubule cytoskeleton is essential for establishing the dynamic intracellular architecture of eukaryotic cells. Microtubules are polar, tube-like polymers consisting of α-/β-tubulin heterodimers. They explore intracellular space by switching between phases of growth and shrinkage. This property, called dynamic instability (Mitchison and Kirschner, 1984), is crucial to microtubule functions (Desai and Mitchison, 1997). In living cells, growing microtubule plus ends provide a transient binding region for a large number of plus-end tracking proteins (+TIPs) that either regulate microtubule dynamics or mediate interactions with other cellular substructures such as the plasma membrane, organelles, or kinetochores of chromosomes in mitosis. +TIPs are therefore crucial for the microtubule cytoskeleton's role in cell division, polarization, and differentiation (Akhmanova and Steinmetz, 2010; Galjart, 2010).

Biochemical in vitro reconstitutions (Bieling et al., 2008, 2007; Dixit et al., 2009; Komarova et al., 2009; Montenegro Gouveia et al., 2010; Zanic et al., 2009; Zimniak et al., 2009) have established that members of one class of evolutionarily conserved +TIPs, the end-binding proteins (EBs), bind autonomously, with their N-terminal calponin homology (CH) domain, to an extended region at the growing microtubule end (Hayashi and Ikura, 2003). They bind to this end region with more than 10-fold higher affinity compared to the microtubule lattice (Maurer et al., 2011) and turn over with fast binding/unbinding kinetics (Bieling et al., 2007). EBs recruit other +TIPs via their C-terminal EB homology domain, making them hubs for a protein interaction network at growing microtubule ends. Some of these recruited proteins have pronounced effects on the dynamic properties of microtubules (Komarova et al., 2002; Montenegro Gouveia et al., 2010), suggesting that misregulation of cellular microtubule dynamics following EB loss of function (Busch and Brunner, 2004; Komarova et al., 2009; Tirnauer et al., 2002) arises primarily from defects in recruitment of these proteins. High-resolution structural studies have provided detailed information about the interaction of the EB homology domain with proteins containing either CAP-Gly domains (Hayashi et al., 2005; Honnappa et al., 2006) or SxIP motifs (Honnappa et al., 2009), but the structural basis of selective EB binding to growing microtubule ends is unknown.

Growth at microtubule ends occurs by the addition of α-/β-tubulin heterodimers with GTP bound in the exchangeable site of β-tubulin. Lattice incorporation triggers GTP hydrolysis so that the vast majority of the microtubule is believed to consist of GDP-tubulin. The microtubule lattice comprises two types of tubulin-tubulin contacts: longitudinal contacts along protofilaments and lateral contacts between the parallel tubulin protofilaments. In cryo-electron microscopy (cryo-EM) images, protofilaments at growing microtubule ends appear more or less straight, either blunt-ended (Mandelkow et al., 1991), flared (Kukulski et al., 2011), or forming gently curved two-dimensional (2D) sheets that close into a straight tube (Chrétien et al., 1995), whereas depolymerizing GDP microtubules always display individual highly curved protofilaments curling outward, “peeling off” from the microtubule end (Chrétien et al., 1995; Mandelkow et al., 1991). This illustrates a topological competition between the spontaneous curvature of GDP protofilaments and the maintenance of lateral contacts necessary for a tubular lattice. Because GDP protofilaments within the lattice are constrained into a metastable straight conformation, the stability of the growing microtubule requires the existence of a capping structure strong enough to keep protofilaments straight, protecting the older part of the microtubule from disassembly. This property is attributed to a layer of GTP-loaded tubulins at the very end of the microtubule, the so-called GTP cap (Carlier et al., 1984; Dimitrov et al., 2008; Mitchison and Kirschner, 1984). The structural origin of the stabilizing effect of the GTP cap is not understood but is mostly attributed to allosteric protofilament straightening as a consequence of nucleotide-dependent longitudinal interactions (Ravelli et al., 2004; Rice et al., 2008).

Several proposals have been made as to which structure EBs bind at growing microtubule ends, including that they recognize 2D tubulin sheets (Vitre et al., 2008). Alternatively, low-resolution EM studies suggested that EBs could bind to the microtubule seam(s) of microtubules polymerized in vitro (des Georges et al., 2008; Sandblad et al., 2006). However, seam binding does not explain the high levels of EB binding to microtubule ends as measured by fluorescence microscopy of microtubules polymerizing in vitro (Maurer et al., 2011). Finally, it was also suggested that EBs might recognize the GTP cap (Zanic et al., 2009). However, the GTP cap is believed to be rather short, probably consisting of only around two tubulins per protofilament (Caplow and Shanks, 1996; Drechsel and Kirschner, 1994). This would be much shorter than the EB binding region, which consists of several hundreds of binding sites (Bieling et al., 2007, 2008; Dixit et al., 2009). Moreover, EBs bind with widely varying affinities to microtubules grown in the presence of different GTP analogs—notably with enhanced affinity for GTPγS microtubules compared to GDP microtubules (Maurer et al., 2011)—suggesting that EBs might instead sense a tubulin conformation that is linked to tubulin's GTPase cycle at growing microtubule ends. The key structural difference between the growing microtubule-end region to which EBs bind with high affinity and the older part of the microtubule is at present still unknown, largely due to a lack of high-resolution structural information.

Here, we set out to address this crucial question using a combination of cryo-EM, subnanometer single-particle reconstruction, and fluorescence microscopy of a reconstituted in vitro system. We show that EBs bind between protofilaments except at the microtubule seam. They contact four tubulins and are ideally positioned to sense the γ-phosphate state of the β-tubulin nucleotide-binding pocket. The EB binding region is characterized by enhanced lateral interprotofilament contacts that protect the microtubule from depolymerization. In combination, our data reveal an unanticipated relationship between the EB binding region and a stabilizing cap structure at microtubule ends crucial for microtubule polymer dynamics.

## Results

### The Mal3 CH Domain Binds to GTPγS Microtubules between Protofilaments except at the Microtubule Seam

**Figure 1:**
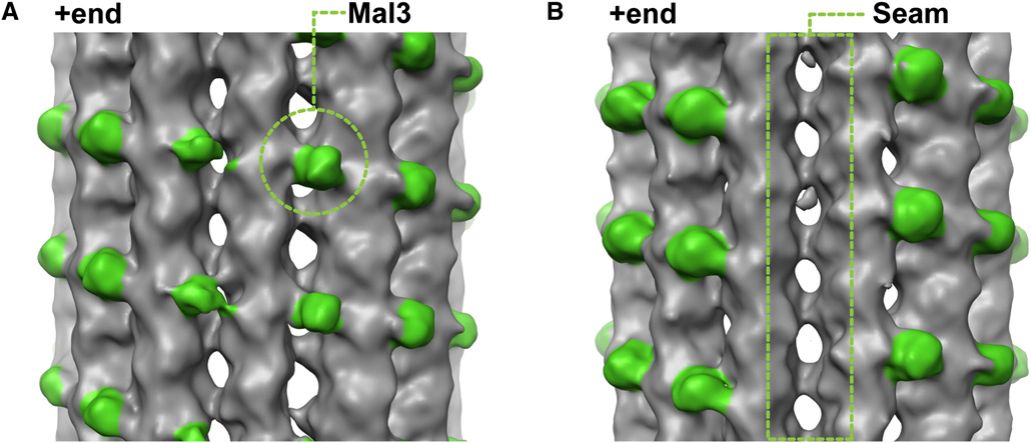
Mal3 Binds between Microtubule Protofilaments except at the Seam (A) 15 Å cryo-EM reconstruction of 13-protofilament Mal3_143_-GTPγS microtubules, displayed with the microtubule plus end oriented up. Mal3_143_ (green) binds the cleft between protofilaments (gray) making B lattice contacts. (B) Same reconstruction as in (A) but rotated 180° around the microtubule axis. The A lattice seam is only marginally occupied. See also Figure S1.

**Figure S1:**
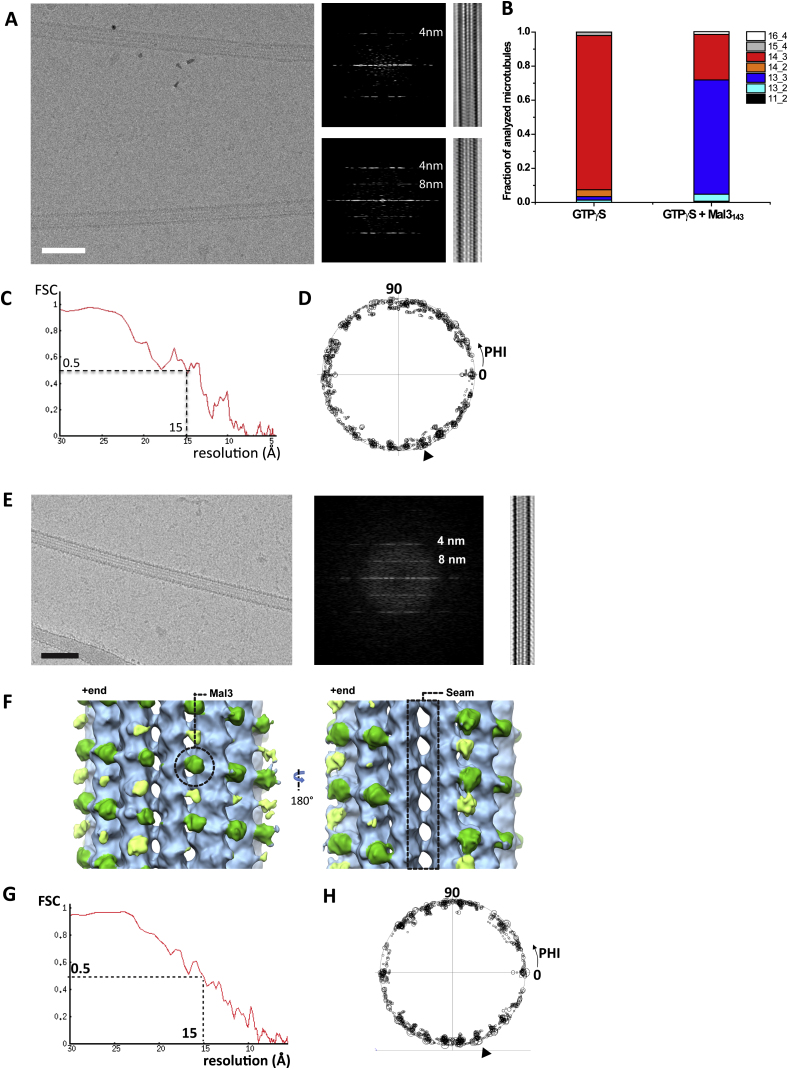
Cryo-EM Imaging and Reconstruction of the Mal3 Microtubule Complex, Related to Figure 1 (A) Raw cryo-EM image (left) of a Mal3_143_-decorated GTPγS microtubule (bottom), grown from quantum dot-labeled GMPCPP microtubule seeds (top), prepared as described previously (Maurer et al., 2011) (scale bar, 50 nm). Only Mal3_143_-decorated GTPγS microtubules were included in our analysis. The 8 nm layerline characteristic of decorated microtubules is strong in power spectra of Mal3_143_-GTPγS-MTs (middle, bottom), and not visible for GMPCPP microtubules (middle, top). A Fourier-filtered Mal3_143_-decorated GTPγS microtubule shows extra densities every other monomer along the protofilaments of the microtubule (right, bottom), which is not seen on the undecorated GMPCPP seed (right, top). (B) Analysis of protofilament number and helix start for GTPγS microtubules grown from GMPCPP seeds in the absence and presence of Mal3_143._ Mal3_143_ promotes the assembly of 13 protofilament 3 start microtubules (13_3, 2% of total population without Mal3_143_, and 68% with Mal3_143_). A small fraction of 2 and 4 start microtubules are also assembled—these microtubules have no seam, reinforcing the preference of Mal3 for B lattice contacts. (C) Fourier shell correlation (FSC) curve of the asymmetric cryo-EM reconstruction of 13 protofilament Mal3_143_-decorated GTPγS microtubules (Figure 1), calculated between two independent half-data sets. The FSC is above 0.5 for resolutions up to 15 Å. (D) Plot of the seam orientations (PHI angles) of the 2D cryo-EM images that went into the reconstruction, showing an isotropic distribution. The orientation of the seam in the reference 3D model is indicated by an arrowhead. (E) Raw cryo-EM image (left) of a Mal3_143_-decorated GDP microtubule. Scale bar, 50 nm. The power spectrum of the Mal3_143_-GDP microtubule displays a strong 8 nm layerline characteristic of decorated microtubules (middle), and corresponding to extra densities every other monomer along the protofilaments of the microtubule seen in the Fourier-filtered image (right). (F) Asymmetric reconstruction of Mal3_143_-GDP microtubules. Mal3_143_ (green) binds the cleft between protofilaments (sky blue) making B lattice contacts and does not bind the A lattice seam. Weaker extra densities between protofilaments (light green) could be due to additional Mal3 CH domains weakly interacting with the interprotofilament valley and/or with other bound CH domains (green). The heterogeneity in binding is consistent with the more than 10-fold lower affinity of Mal3 for the GDP lattice compared with the growing end region (Maurer et al., 2011). Averaging B lattice contacts resulted in a relatively noisy map with lower resolution compared with the symmetrised Mal3_143_-GTPγS map (not shown). (G) FSC curve of the asymmetric cryo-EM reconstruction of 13 protofilament Mal3_143_-decorated GDP microtubules, calculated between two independent half-data sets. The FSC is above 0.5 for resolutions up to 15 Å. (H) Plot of the seam orientations (PHI angles) of the 2D cryo-EM images that went into the reconstruction, showing an isotropic distribution. The orientation of the seam in the reference 3D model is indicated by an arrowhead.

We used cryo-EM to determine how the CH domain of Mal3, the fission yeast EB, binds to GTPγS microtubules, which were previously shown to be static mimics of growing microtubule ends (Maurer et al., 2011). We found that Mal3 favors the assembly of GTPγS-tubulin into microtubules with mostly 13 protofilaments (Figure S1B available online), consistent with a previous study of EB1 in presence of GTP-tubulin (Vitre et al., 2008). Three-dimensional (3D) reconstruction from segments of 13 protofilament GTPγS microtubules decorated with an N-terminal fragment of Mal3 containing its CH domain (Mal3_143_) generated an asymmetric structure with 15 Å resolution (Figures 1, S1C, and S1D). Mal3_143_ binds regularly between neighboring protofilaments (B lattice, 12 such contacts per 13 protofilament microtubule) except along the seam (the single A lattice contact between protofilaments) of GTPγS microtubules (Figure 1B). This selectivity suggests a highly specific binding site. The longitudinal distance between bound CH domains is 8 nm (corresponding to one tubulin heterodimer), resulting in a stoichiometry of 12 CH domains per 13 tubulin dimers. This is in good agreement with the recently reported approximately stoichiometric binding measured by fluorescence microscopy (Maurer et al., 2011). A similar pattern of binding—including absence of interaction at the seam—was seen for Mal3_143_ on GDP microtubules (Figures S1E–S1H). This is in contrast to previous reports (des Georges et al., 2008; Sandblad et al., 2006) (see Discussion). Our data suggest that the affinity difference of Mal3 binding to growing microtubule ends compared to older parts of the microtubule lattice (Maurer et al., 2011) is not a result of different binding positions nor of dramatic A-to-B lattice transitions within the microtubule but rather a consequence of conformational rearrangements within its binding site.

### Mal3 CH Domain Binds at the Corner of Four Tubulin Dimers

**Figure 2:**
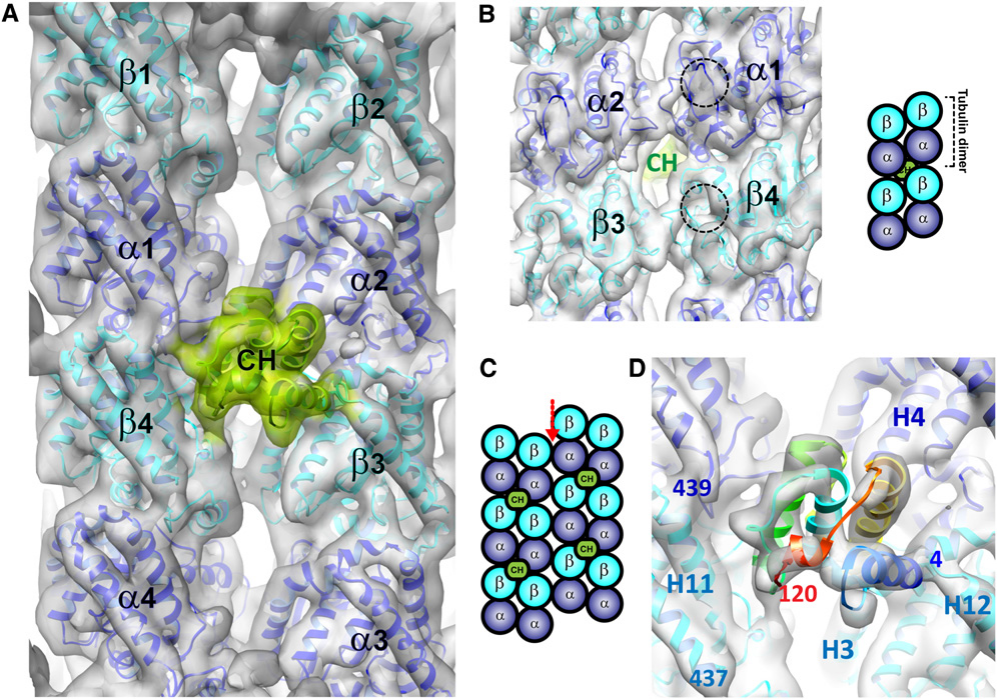
A Pseudoatomic Model of the EB Microtubule-Binding Site (A) 8.6 Å reconstruction of the Mal3_143_-microtubule interface docked with atomic structures of tubulin (Fourniol et al., 2010) (cryo-EM map, gray surface; 2XRP.pdb; α in blue, β in cyan ribbons) and with a homology model of the Mal3 CH domain (see Experimental Procedures; Slep and Vale, 2007) (map, green surface; Mal3 CH atomic model, green ribbons). (B) Lumenal surface of the reconstruction shown in (A). Dotted circles highlight a region where tubulin monomers clearly differ in the EM map, delineated by the M loop, H6-H7 loop, and helix H7: the EM maps show an empty taxol-binding pocket in β-tubulin (Nogales et al., 1999), whereas an extra density is seen in α-tubulin, which corresponds to an insertion in loop S8-S9 specific to α-tubulin. This enables unambiguous assignment of the α- and β- tubulin densities. A schematic of this lumenal view shows the localization of Mal3 CH domain at the corner of four tubulin heterodimers. (C) Schematic view of the outer microtubule surface illustrating that the Mal3-binding interface does not exist at the seam (red arrow). (D) Close-up of the interface (map rendered at a higher threshold compared with B, gray surface; Mal3 CH model, rainbow-colored ribbons). The residue number of the boundaries of the Mal3 CH domain and the C termini of α- and β-tubulin are labeled, as are tubulin helices α2-H4, β3-H3, β3-H12, and β4-H11. See also Figure S2 and Movie S1.

**Figure S2:**
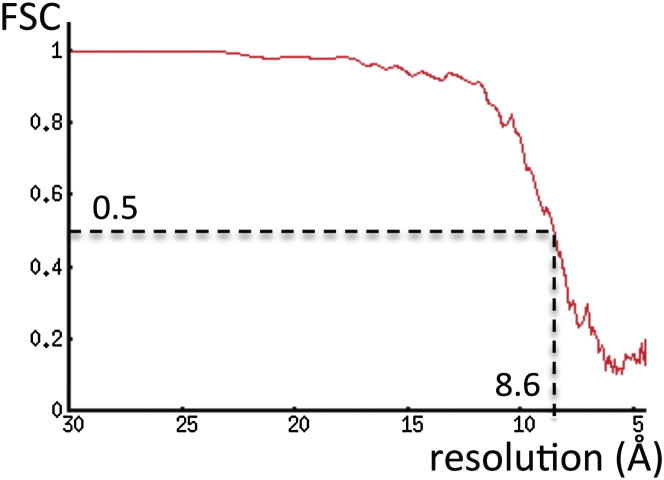
FSC Curve of the Symmetrized Reconstruction of the Mal3_143_-Decorated GTPγS Microtubule Interface, Related to Figure 2 Calculated between two independent half-data sets. The FSC is above 0.5 for resolutions up to 8.6 Å.

Averaging B lattice contacts of the Mal3 CH domain on GTPγS microtubules produced an 8.6 Å resolution reconstruction in which the secondary structural elements of tubulin and the CH domain of Mal3 are clearly resolved (Figure 2A, gray and green envelope, respectively; Figure S2; Movie S1). This subnanometer resolution allowed α- and β-tubulins to be distinguished unambiguously (Figure 2B) and a pseudoatomic model of the Mal3-binding site to be generated (Figure 2D). The Mal3 CH domain contacts four different tubulin dimers (Figures 2A and 2C), providing an explanation for how the CH domain distinguishes between B lattice contacts and the microtubule seam: the binding site is formed by two adjacent α-tubulin contacts (toward the microtubule plus end) and two adjacent β-tubulin contacts (toward the minus end). This configuration is not present at the seam where lateral α-β contacts exist (Figure 2C). In binding between protofilaments, the EB footprint is distinct from that of microtubule-based motors kinesin and dynein, which step along the protofilament ridge (Mizuno et al., 2004).

**Figure 3:**
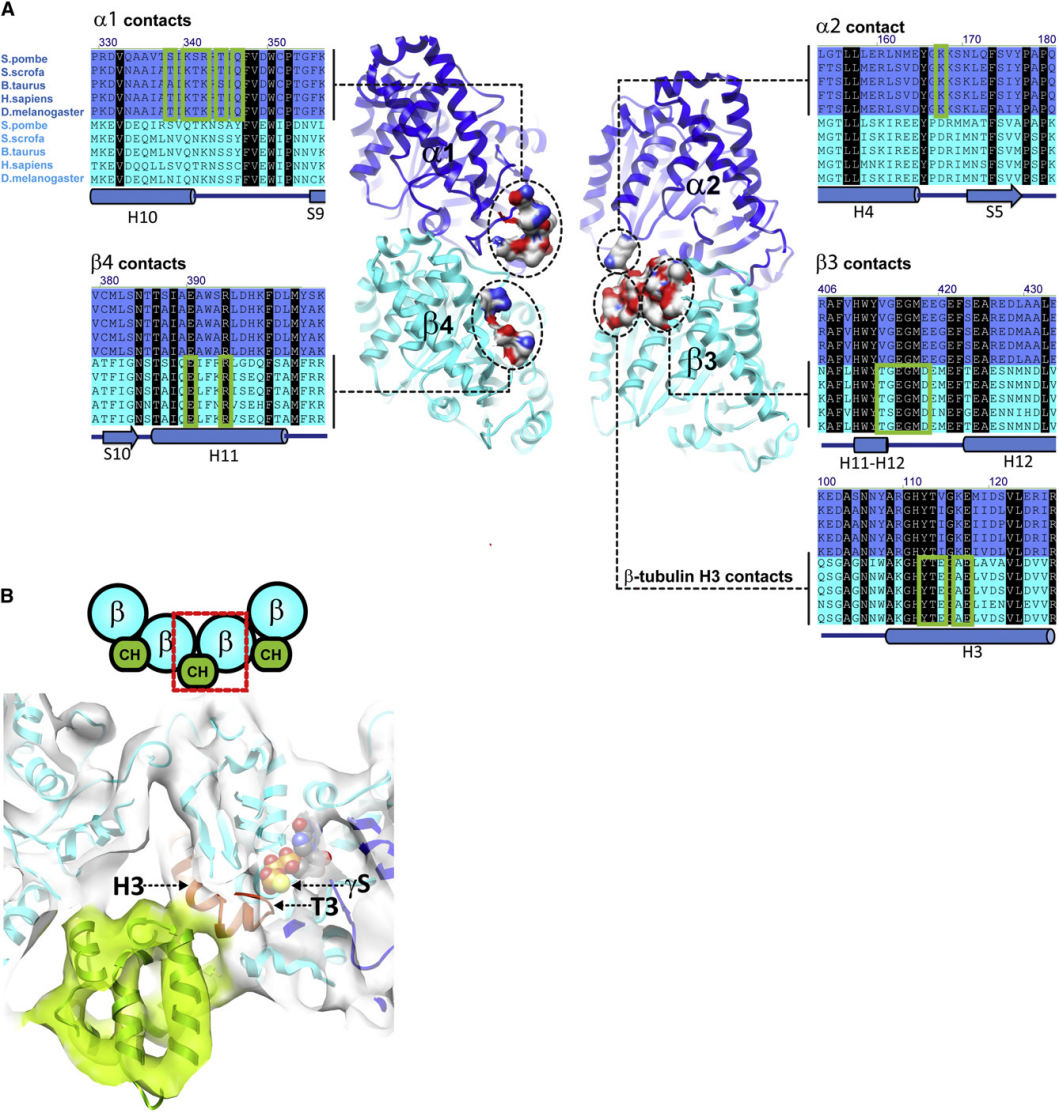
Structural Features of the GTPγS Microtubule Recognized by the CH Domain (A) Front view of the four tubulin monomers (α in blue, β in cyan) contacted by the Mal3 CH domain, with tubulin residues within 5 Å of Mal3 displayed as a molecular surface with colored heteroatoms. Sections of sequence alignments of tubulins from five different species (α in blue, β in cyan) covering the Mal3 contact regions (green boxes). Residues conserved between α- and β-tubulin are shown white on black. Secondary structures of α- and β-tubulin (1JFF-A) are depicted below. (B) Cross-section of the Mal3-GTPγS microtubule map at the interdimer interface, seen from the plus end. GTPγS (spacefill) was docked in the β-tubulin nucleotide pocket. The γ-S-phosphate group is coordinated by the T3 loop (magenta) at the N-terminal extremity of the β-tubulin H3 helix (magenta) contacted by the EB CH domain (green). See also Figure S6 and Movie S1.

**Figure 4:**
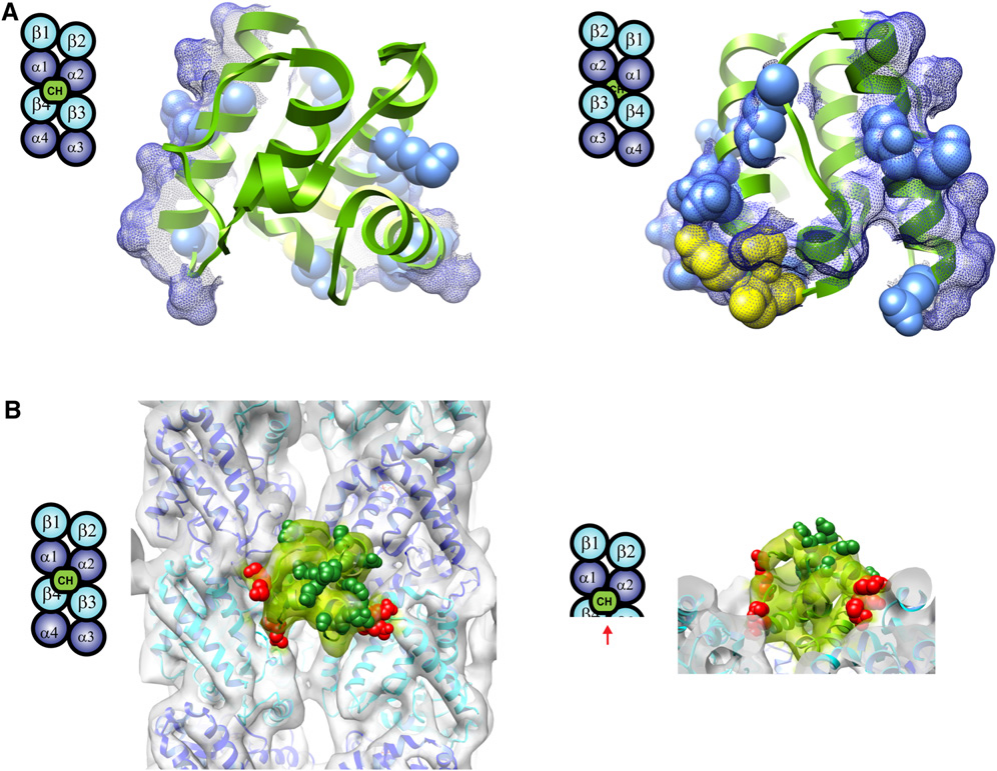
EB Residues Important for Growing Microtubule-End Recognition (A) Two views of conserved EB surface residues at the microtubule interface. The blue mesh depicts the surface of Mal3 CH domain residues found to be <5 Å away from tubulin residues in the pseudoatomic model. Almost all of the conserved CH domain surface residues found in the five EBs (spacefill atoms) form part of the contact surface. An especially large fraction of conserved residues (yellow spacefill) contact the β-tubulin H3 helix, whereas other conserved residues (blue spacefill) are part of the other tubulin contacts. (B) The identified Mal3-GTPγS microtubule interface provides a structural explanation for previous mutagenesis results obtained with EB1 (Slep and Vale, 2007) (left, front view; right, end-on view from the minus end). Previous mutations shown to disrupt plus-end tracking of EB1 in cells correspond to amino acids contacting the microtubule surface (red spacefill). Our structure shows that these patches are part of contact sites between the CH domain and β3- or β4-tubulin. In contrast, mutations without a noticeable effect are distant from the microtubule surface (green spacefill). See also Figure S3.

**Figure S3:**
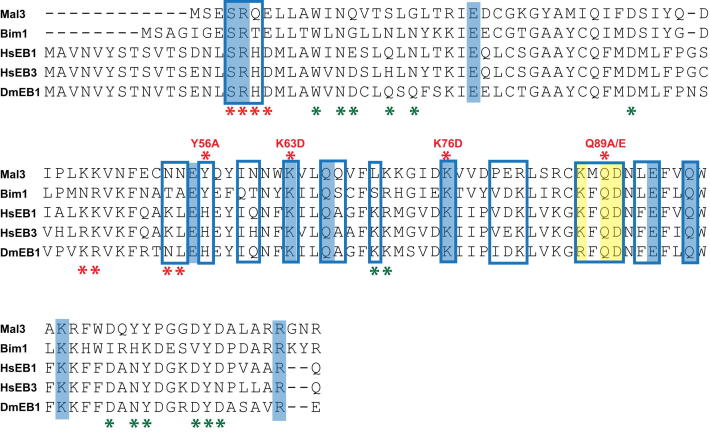
Sequence Alignment of the CH Domains of EBs, Related to Figure 4 Conserved residues (highlighted in blue; and in yellow for the β-tubulin H3 helix contacts) correlate well with the microtubule-binding interface (blue frames, Mal3 CH domain residues found to be <5 Å away from tubulin residues in the pseudoatomic model). Mutations are indicated by asterisks above (this study) or under (previous study; Slep and Vale, 2007) the alignment with the colors indicating disruption of end tracking (red) or no influence on end tracking (green).

Mal3_143_ contact sites identified in our pseudoatomic model are conserved within α- and within β-tubulins from different species but not between α- and β-tubulins, explaining why the CH domain can distinguish between the two subunits of the tubulin heterodimer (Figure 3A). In addition, Mal3_143_ residues close to the microtubule surface (Figure 4A, blue mesh) match closely to conserved residues on the surface of its CH domain (Figure 4A, blue and yellow spacefill residues). These data strongly suggest that the structural basis of the recognition of growing microtubule ends by EBs is conserved. Our structure also shows that the microtubule-binding interface of the CH domain is much more extensive than suggested by a previous in vivo mutagenesis study (Slep and Vale, 2007), where deleterious mutations lie at two of the identified Mal3-tubulin interfaces (on β3- and β4-tubulin) (Figure 4B, red spacefill; Figure S3, red asterisks below alignment), whereas silent mutations lie away from these regions (Figure 4B, green spacefill; Figure S3, green asterisks below alignment). In particular, our model reveals that as well as additional contacts with two α-tubulins (α1 and α2), Mal3_143_ also contacts β3-tubulin at lower radius on the H3 helix, which, strikingly, is directly connected to the exchangeable nucleotide site (E site, Figure 3B).

**Figure 5:**
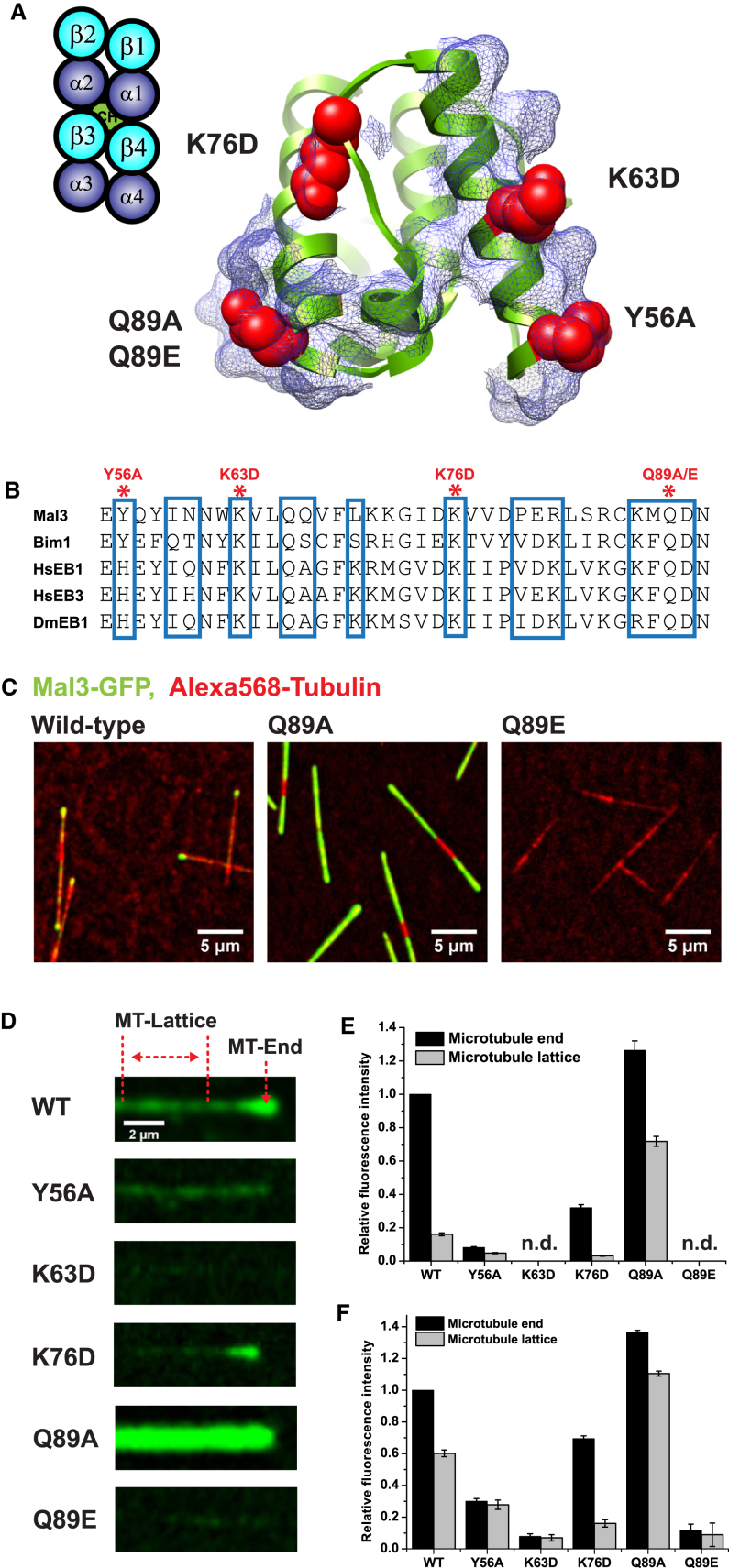
Validation of EB-Tubulin Contact Sites using Reconstituted Dynamic Microtubule-End Tracking (A) Localization of single mutated residues on the Mal3 microtubule-binding surface: residues chosen for mutations (red) in the context of all Mal3 residues within 5 Å of tubulin (rendered as a blue mesh). (B) Extract of a sequence alignment of different EBs: Mal3 residues contacting the microtubule (blue boxes) and residues that were mutated in this study (red asterisks). (C) TIRF microscopy images of Alexa 568-labeled microtubules (red) grown in the presence of GTP and wild-type (WT) or mutant Mal3-GFP (green) as indicated. (D) TIRF microscopy images illustrating the differences between the Mal3-GFP fluorescence signal (green) for WT and the different mutants on single microtubules. Imaging conditions and display settings are identical for all six experiments. The regions used to measure Mal3-GFP intensity at the microtubule end and on the lattice are indicated by red lines. (E and F) Quantification of Mal3-GFP intensities at the microtubule ends and on the lattice. Error bars represent standard error of the mean (SEM). (E) Mean intensities relative to the mean WT intensity at growing microtubule ends are shown. Concentrations were 30 nM Mal3-GFP, 22 μM tubulin, and 1 mM GTP. (F) Same as (E) with 300 nM Mal3-GFP. See also Figure S4 and Movie S2.

**Figure S4:**
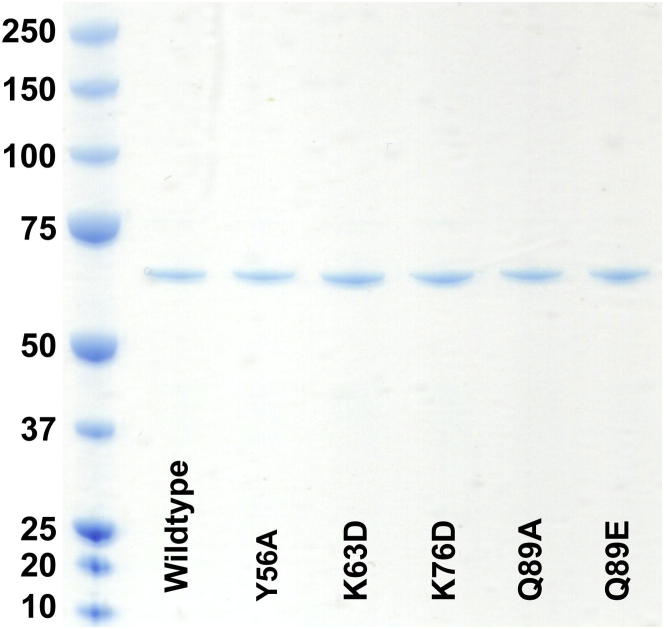
Coomassie-Stained SDS-PAGE of Mal3-GFP Proteins Used for This Study, Related to Figure 5 Molecular weights are in kDa.

In order to test whether our model derived from monomeric Mal3 on GTPγS microtubules reflects the behavior of full-length EB end tracking on dynamic microtubules, we selected conserved Mal3 residues from the tubulin contact sites (Figures 5A and 5B), produced the corresponding single-point mutants in full-length dimeric Mal3-GFP (Figure S4), and tested their ability to track the ends of microtubules grown with GTP in vitro. Using total internal reflection fluorescence (TIRF) microscopy, we found that most selected Mal3 mutants either abolished or severely weakened end tracking (Figure 5; Movie S2), validating our model. Inverting charges at either of the contacts with α-tubulin (Mal3K63D-GFP and Mal3K76D-GFP) resulted in weaker binding of Mal3 (Figures 5D–5F), whereas the Mal3Y56A-GFP mutant (β4-tubulin contact) also strongly reduced Mal3 binding (Figures 5D–5F), indicating that the exact charge and geometry of these interfaces are important for interaction of Mal3 with the microtubule. However, when we investigated the importance of the β3-tubulin H3 contact, we found that the Mal3Q89E-GFP mutant had strongly impaired microtubule-end tracking, whereas Mal3Q89A-GFP bound with higher affinity to the entire microtubule than the wild-type (WT) (Figures 5C–5F; Movie S2). A recent in vivo study reported a similar enhancement of lattice binding and loss of end tracking for a Mal3 mutation at the same position (Mal3Q89R; Iimori et al., 2012). This finding together with our in vitro mutational analysis of the EB-H3 helix interface underlines the importance of this contact for EB microtubule-end tracking.

**Figure 6:**
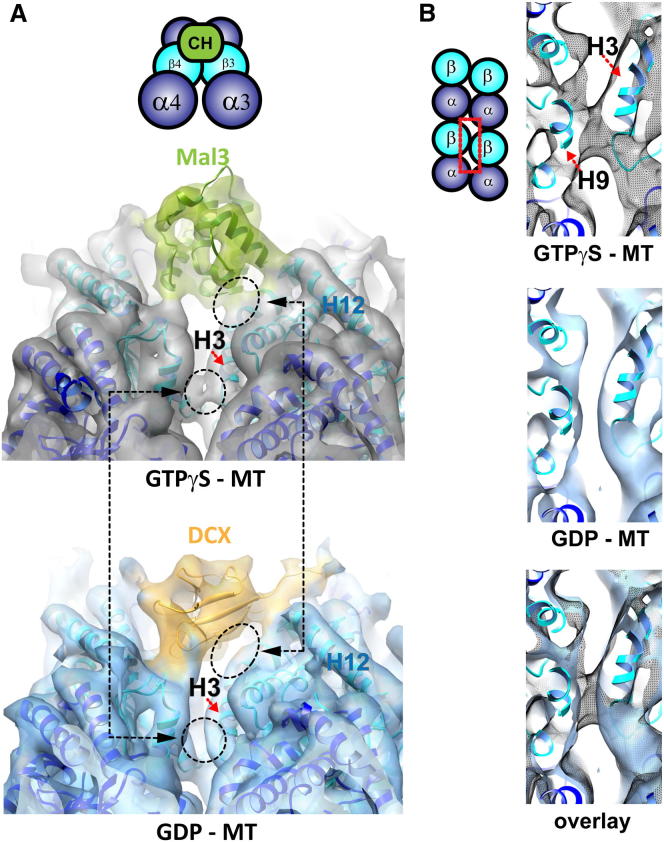
Additional Lateral Contacts Are Observed in the GTPγS Lattice (A) Comparison of the cryo-EM structures of a Mal3-GTPγS microtubule (8.6 Å resolution) and a GDP microtubule stabilized by doublecortin (Fourniol et al., 2010) (DCX; EMDB ID 1788; 8.2 Å resolution) viewed from the microtubule minus end. An extra interprotofilament contact involving β-tubulin helix H3 (dashed circle) is present in the GTPγS microtubule structure but not in the GDP microtubule structure. (B) Close-up views of the GTPγS and GDP microtubule cryo-EM structures described in (A), focused on the area involved in the additional β-tubulin lateral contact. The two cryo-EM maps are displayed with an equivalent threshold, representative of the whole protein complex volume. See also Figure S5 and Movie S1.

The only other protein known to bind to the corner of four tubulin heterodimers is doublecortin, a microtubule-stabilizing protein that is unrelated to EBs (Fourniol et al., 2010). Together, these two proteins define a polymer-specific binding mode characterized by bridging of microtubule protofilaments. Comparison of the subnanometer models of doublecortin bound to GDP microtubules and Mal3_143_ bound to GTPγS microtubules reveals, however, that the contacts between the corners of the four tubulin monomers and each of these proteins are not identical and, in particular, the contact with the β-tubulin H3 helix appears to be unique to EBs (Figure 6A).

### Enhanced Lateral Interprotofilament Contacts Characterize the Microtubule Structure Recognized by the EB CH Domain

**Figure S5:**
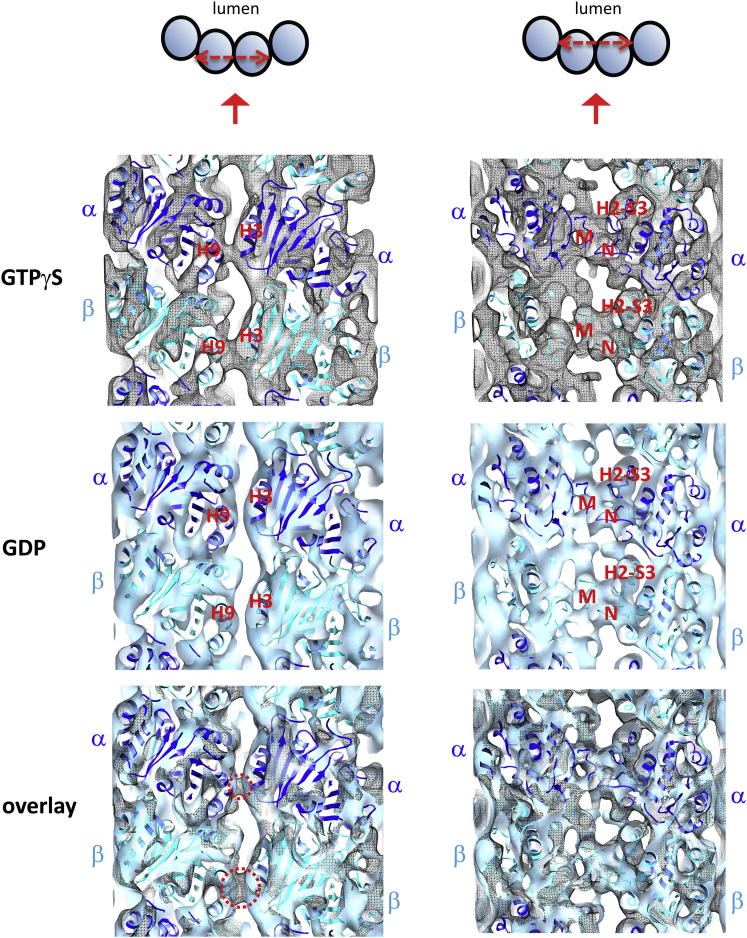
Comparison of Lateral Tubulin-Tubulin B Lattice Contacts in GTPγS and GDP Lattices, Related to Figure 6 Comparison of the cryo-EM maps of Mal3-decorated GTPγS microtubules (top, gray mesh, 8.6 Å resolution, see also Figure 6) and GDP microtubules stabilized by doublecortin (middle, blue surface; EMDB 1788; Fourniol et al., 2010; 8.2 Å resolution). Longitudinal sections of the EM map illustrating the two layers of tubulin-tubulin lateral contacts: additional H3-H9 contacts are observed only in GTPγS microtubules (left), whereas the conserved contacts involving the M loop, N loop, and H2-S3 loops of tubulin (Sui and Downing, 2010) are observed in both GTPγS and GDP microtubules (right). Interestingly, although the additional H3-H9 contacts in GTPγS microtubules appear to be more pronounced for neighboring β-tubulin contacts, they are also visible between neighboring α-tubulins, suggesting that concerted conformational changes take place in the GTPγS lattice-incorporated tubulin heterodimers. The overlays (bottom) illustrate the good agreement between the two density maps displayed with an equivalent contouring level representative of the whole protein complex volume (GTPγS map rendered at a threshold of 1.68 σ; DCX-GDP map rendered at a threshold of 0.78 σ), and highlights the differences in density due to H3-H9 contacts (circled, the β-β contact extends over ∼10 Å, the α-α contact over ∼5 Å).

In Mal3_143_-decorated GTPγS microtubules, as in all subnanometer microtubule reconstructions reported to date (Fourniol et al., 2010; Li et al., 2002; Sui and Downing, 2010), interprotofilament lateral contacts that involve secondary structure elements facing the microtubule lumen are observed: the M loop (S7-H9) of one subunit contacts the N loop (H1-S2) and H2-S3 loop of the neighboring tubulin subunit (Figure S5). However, strikingly, we also observed an enhanced layer of lateral contacts at higher radius in GTPγS microtubules (Figures 6B and S5). These enhanced lateral contacts involve tubulin helices H3 that adjoin H9 of neighboring tubulins, presumably because of a structural change—possibly a positional shift—in the H3 helix of β-tubulin in the GTPγS microtubule. This might be part of the structural alteration that is sensed by EBs. A similar but smaller lateral contact between α-tubulins (Figure S5) is likely to result from cooperative conformational rearrangements within the lattice. The importance of the H3 contacts in our structure is consistent with alanine scanning mutagenesis in yeast, showing that mutations in α- and β-tubulin H3 cause temperature sensitivity (Reijo et al., 1994; Richards et al., 2000). The structural change in the β-tubulin H3 helix could be triggered by nucleotide hydrolysis, sensed by H3 via loop T3, which might be mimicked by the presence of a bulky group such as the γ-S-phosphate (or BeF3^−^; Maurer et al., 2011) occupying the γ-phosphate-binding pocket. EBs appear therefore to be optimally positioned to sense nucleotide hydrolysis-dependent structural changes in the microtubule lattice.

### The EB Binding Region Is a Stabilizing Structural Cap Protecting the Microtubule from Depolymerization

**Figure 7:**
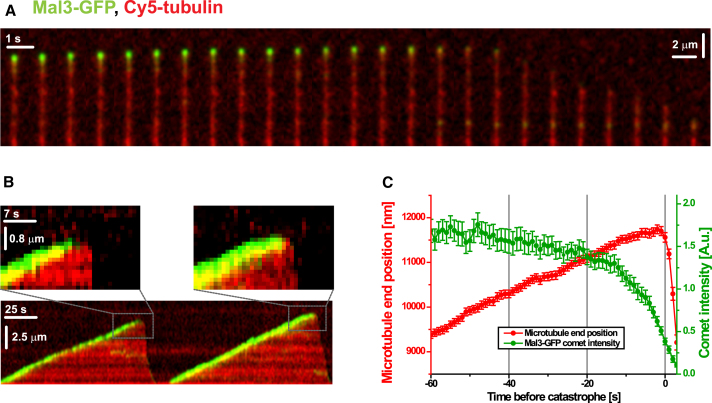
The EB Binding Region Disappears before Catastrophe Occurs (A) Time series of Mal3-GFP on a microtubule grown in GTP, imaged by TIRF microscopy. The image sequence depicts a typical fluorescence time course of Mal3-GFP at a microtubule end at the transition from growth to shrinkage. Mal3-GFP is shown in green, Cy5-labeled microtubules in red. (B) Kymograph of one microtubule showing two consecutive growth and catastrophe episodes. Color code is as in (A). The periods directly before and after a catastrophe are magnified in the insets. (C) Plot of the averaged normalized Mal3-GFP comet intensity (green) and the averaged relative microtubule-end position (red) as a function of time prior to catastrophe (average of 62 catastrophe events). The error bars are SEM; Mal3-GFP concentration was 60 nM. For details, see the Experimental Procedures.

The enhanced interprotofilament contacts recognized by EBs could have a potentially crucial role in stabilizing the lattice of dynamic microtubules. If so, we would predict that such contacts are lost before microtubule depolymerization, thereby reducing EB affinity at microtubule ends prior to catastrophe. We tested this hypothesis by recording dynamic microtubules in vitro in the presence of full-length dimeric Mal3-GFP by TIRF microscopy. We measured the Mal3-GFP comet intensity as a read-out for the presence of the enhanced interprotofilament contacts and asked whether the Mal3-GFP comets disappear before catastrophe. This was indeed the case (Figures 7A and 7B). The average comet intensity began to decay several seconds before catastrophe and was strongly reduced at the moment of catastrophe (Figure 7C). This observation suggests two possible interpretations: either the loss of Mal3 or a conformational transformation within the microtubule lattice leading to the loss of most of the EB binding region causes a catastrophe. It is unlikely that the loss of Mal3 from the microtubule end triggers catastrophe because in vitro the addition of EBs is known to increase the catastrophe frequency under the conditions used here (Bieling et al., 2007; Komarova et al., 2009). Thus, we can define the extended high-affinity EB binding region as a stabilizing zone at growing microtubule ends. The loss of this zone appears to trigger depolymerization. Surprisingly, EBs were found to decrease the lifetime of their own high-affinity binding sites and hence the size of the protective zone at microtubule ends (Maurer et al., 2011). Within the framework of the model of the extended protective structural cap, this provides a direct explanation for the catastrophe-promoting effect of EBs in vitro.

## Discussion

Our data provide an explanation for the structural basis of microtubule-end tracking by EBs, a conserved class of proteins forming the core of a dynamic interaction network at growing microtubule ends. EBs recognize a structural feature on the microtubule surface that is intimately linked to dynamic instability. They distinguish between the growing microtubule-end region and the older part of the microtubule by sensing a tubulin conformation that stabilizes the microtubule end and that transforms with time into the metastable GDP lattice.

Our subnanometer reconstruction shows that the EB CH domain bridges neighboring protofilaments by precisely contacting four tubulin dimers arranged in the B lattice configuration, characteristic for almost all interprotofilament contacts in the microtubule. This binding mode explains the large number of EB binding sites at growing microtubule ends (Maurer et al., 2011) and emphasizes that the microtubule-end region can serve as a binding platform for many hundreds of +TIP molecules. The contacts between the EB CH domain and the microtubule are evolutionarily conserved, reflecting the conservation of the phenomenon of microtubule-end tracking and also explaining why proteins from different species can be used interchangeably in reconstituted in vitro assays (Bieling et al., 2007; Zimniak et al., 2009) and in in vivo rescue experiments (Browning et al., 2003). It seems likely that EBs would not bind at the very ends of growing microtubules due to incomplete formation of their binding sites. In contrast, major effectors of dynamic instability such as destabilizing kinesins (Helenius et al., 2006) and other motors are expected to be able to localize to the very ends of microtubules because they bind on the ridge of single protofilaments (Mizuno et al., 2004).

Our data also explain why EBs do not bind to the microtubule seam, where lateral α-tubulin/β-tubulin contacts form a discontinuity in the tubulin lattice. Previous, contradictory suggestions that Mal3 selectively binds to the seam were based on lower-resolution data from considerably smaller datasets and from more heterogeneous GDP microtubule samples (des Georges et al., 2008; Sandblad et al., 2006). We observed that, in comparison to GTPγS microtubules, GDP microtubules in the presence of saturating concentrations of Mal3_143_ produced a less homogenous dataset, presumably due to low-affinity binding (Maurer et al., 2011). Although this prevented us from obtaining a reliable subnanometer reconstruction for decorated GDP microtubules, our low-resolution reconstruction nevertheless clearly shows that, independent of the tubulin-bound nucleotide, EBs do not bind to the microtubule seam. Furthermore, seam binding would only be consistent with measured binding stoichiometries of EBs (des Georges et al., 2008; Maurer et al., 2011) if it is assumed that the entire growing microtubule end is formed from A lattice (seam) contacts. This is, however, unlikely because the GDP microtubule lattice is known to consist of mostly B lattice contacts both in vitro and in vivo (McIntosh et al., 2009). Therefore, large-scale rearrangements of interprotofilament contacts would be required as the microtubule matures if the end region consisted mostly of A lattice contacts. In contrast, our model links the higher affinity of EBs for the growing microtubule-end region to a structural change within B lattice-incorporated tubulin that is linked to its GTPase cycle.

Our reconstruction of the GTPγS microtubule offers a subnanometer view of the structure of a microtubule lattice with a nucleotide in the exchangeable site that occupies the γ-phosphate-binding site. Early pseudoatomic models built from low-resolution (14–20 Å) cryo-EM reconstructions of taxol-stabilized GDP microtubules (Nogales et al., 1999) and GMPCPP microtubules (Meurer-Grob et al., 2001) suggested tubulin regions potentially involved in the formation of extra lateral contacts at the GTP cap. But the involvement of these regions was subsequently questioned when a subnanometer resolution structure of GDP+taxol microtubules became available (Li et al., 2002). Reconstructions with resolutions of ∼9 Å or better are essential to reveal the subtle, monomer-specific conformational changes caused by tubulin-bound nucleotides (Figures 6 and S5). In the future, it will be interesting to compare other subnanometer microtubule structures bound with various nucleotides. Importantly, in our 8.6 Å resolution reconstruction of GTPγS microtubules, the conformation of the β-tubulin H3 helix—the N-terminal extremity of which forms the boundary of the exchangeable GTP-binding site of the heterodimer (Figure 3B)—enables the formation of enhanced lateral contacts between the protofilaments. Because lateral contacts have to break when protofilaments peel off from a depolymerizing microtubule, such strengthened lateral contacts could provide an explanation for the apparent protection of the growing microtubule end from catastrophe by the EB binding region both in vitro (Figure 7) and in vivo (Busch and Brunner, 2004). Thus, our finding that the EB binding region is a stabilizing structural cap suggests that the phenomena of microtubule dynamic instability and +TIP activity are intimately linked.

Structural protection of dynamic microtubule ends has traditionally been assigned to the short GTP cap. Mechanistically, this has been attributed to a straighter conformation of tubulin in this nucleotide state, which is more compatible with the formation of lateral contacts in the microtubule lattice (Desai and Mitchison, 1997). However, the extent to which GTP-stimulated tubulin straightening contributes to microtubule stabilization during growth is still under debate (Nawrotek et al., 2011; Ravelli et al., 2004; Rice et al., 2008; Wang and Nogales, 2005). Our results suggest instead that enhanced lateral contacts within an extended region of the lattice might play a major role in stabilization at the growing microtubule end. As the rate of tubulin incorporation at the growing end is subject to stochastic fluctuations (Gardner et al., 2011; Schek et al., 2007), a long stabilizing cap would provide a “buffer“ zone to help maintain growth over long distances.

Our subnanometer structure shows that EBs are well placed to detect GTP hydrolysis-induced conformational changes in the growing microtubule-end region. The finding that EBs bind considerably more strongly to GTPγS and [GDP + BeF3^−^] microtubules (Maurer et al., 2011) than to GMPCPP microtubules (which is considered the bona fide GTP analog for microtubules; Hyman et al., 1992) indicates that the GTP cap and the EB binding region at growing microtubule ends are, in fact, different and that EBs might rather bind with high affinity to a conformation induced by GTP hydrolysis. In this model, the high-affinity EB binding state of tubulin has a lifetime of several seconds (Bieling et al., 2007) before it slowly transforms to the conformational GDP state, perhaps reflecting the structural plasticity of the microtubule lattice (Kueh and Mitchison, 2009). Because the conformational transition is slow, the binding region of EBs is rather extended, consisting of several hundreds of tubulins and giving rise to the comet-shaped appearance in fluorescence microscopy images. The EBs themselves bind very dynamically to this region with dwell times considerably shorter than a second (Bieling et al., 2007, 2008; Dixit et al., 2009; Montenegro Gouveia et al., 2010) so that they can respond to the underlying distribution of the high-affinity binding sites.

**Figure S6:**
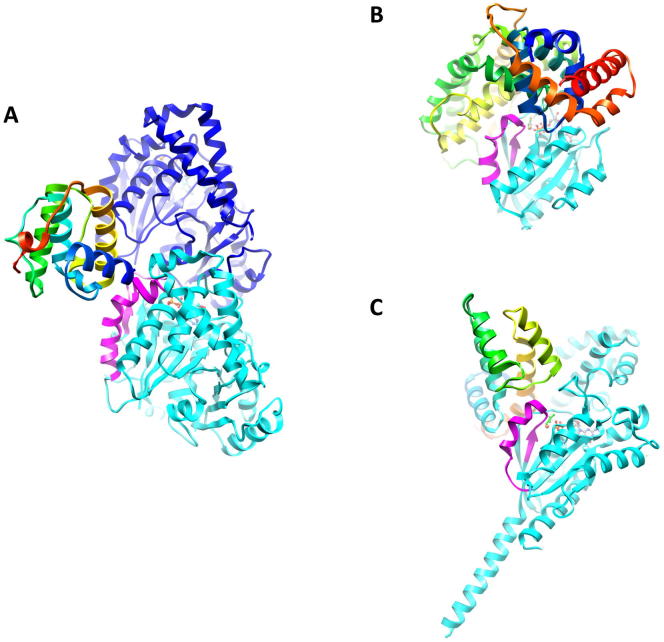
Comparison between the EB-Tubulin and G-Protein-GAP Complexes, Related to Figure 3 (A) Pseudoatomic model of Mal3-microtubule interaction (Mal3 CH model, rainbow-colored ribbons) showing α2-tubulin (blue), β3-tubulin (cyan), and GTPγS (ball-and-stick model). The putative switch II T3-H3 motif of β-tubulin is highlighted in magenta. (B) Structure of small G protein Ras (PDB 1WQ1) (cyan) complexed with Mg.GDP.AlF_3_ (ball and stick) bound to RasGAP (rainbow-colored ribbons) (Scheffzek et al., 1997). The Ras switch II motif is highlighted in magenta. (C) Structure of the heterotrimeric G protein G_iα1_ (PDB 1AGR) (cyan) complexed with Mg.GDP.AlF_4_ (ball and stick) bound to its GAP RGS4 (rainbow-colored ribbons) (Tesmer et al., 1997). The G_iα1_ switch II motif is highlighted in magenta. Whereas the GAPs of small G proteins such as Ras contribute catalytic residues to the GTPase reaction, the GAPs of heterotrimeric G proteins act indirectly by stabilizing the switch region of the catalytic G_α_ subunit, thereby stabilizing the catalytic transition state of GTP hydrolysis. The comparable binding site of EBs with respect to the β-tubulin catalytic site suggests that EBs could play an equivalent role.

At high concentrations, Mal3 has been observed to reduce the lifetime of its own binding site—i.e., the extended stabilizing region at growing microtubule ends—by up to a factor of two (Maurer et al., 2011). In this context it is interesting to note that the interaction between EBs and GTPγS microtubules is structurally reminiscent of the GTPase-activating proteins (GAPs) of G proteins, which are molecular switches in cell-signaling circuits (Figure S6) (Vetter and Wittinghofer, 2001). In particular, the GAPs of heterotrimeric G proteins stimulate the basal GTPase activity of their cognate Gα protein. Thus, β-tubulin helix H3 might be functionally equivalent to the switch II helix in other GTPases, as previously suggested (Nogales et al., 1999). This structural analogy supports the possibility that EBs might recognize a conformational state of the microtubule lattice induced by or during GTP hydrolysis. Future studies will be aimed at testing this intriguing hypothesis.

At physiological concentrations, which have been estimated to be in the range of several 10s of nM (Katsuki et al., 2009; Tirnauer et al., 2002), EBs have a catastrophe-stimulating effect in vitro (Bieling et al., 2007; Komarova et al., 2009). More complex effects arise from perturbation of EB function in vivo, including, surprisingly, the destabilization of microtubules upon loss of EB function (Busch and Brunner, 2004; Komarova et al., 2009; Tirnauer et al., 2002). This cannot be explained by the direct effect EBs have on microtubules as revealed by in vitro experiments. Targeting of other +TIPs to the microtubule-end region by EBs can also not fully explain the microtubule-stabilizing effects that EBs have in vivo, suggesting that presently unknown additional activities contribute (Komarova et al., 2009). Our current findings provide the mechanistic framework to investigate these effects further.

### Conclusion

Our subnanometer structural data define the binding mode of EBs at growing microtubule ends. EBs bridge protofilaments at the corners of four tubulin subunits, except at the seam. EBs are ideally positioned to sense conformational changes induced by GTP hydrolysis. We propose that a structural rearrangement of the β-tubulin H3 helix results in two topological changes within the microtubule: first, more pronounced lateral contacts between neighboring tubulins, and second, a concomitant establishment of a high-affinity binding site for the CH domain of EBs. EBs recognize an extended structural cap that stabilizes the growing microtubule end, linking the phenomenon of microtubule plus-end tracking to the requirement of a structure protecting the growing microtubule from depolymerization. By linking the recognition of the end region with combinatorial recruitment of other proteins, EBs turn the microtubule end into a mobile port and provide anchoring sites for a variety of plus-end tracking proteins, thereby adding further layers of cellular functionality to microtubule ends.

## Experimental Procedures

### Protein Biochemistry

Proteins were purified, and single-point mutants of full-length Mal3-GFP for fluorescence imaging were produced using standard methods and as described (Maurer et al., 2011).

### Cryo-EM Sample Preparation

GTPγS microtubules were decorated with monomeric Mal3_143_ for EM as described (Maurer et al., 2011). Briefly, GTPγS microtubules were grown from quantum dot-labeled GMPCPP seeds in the presence of 2 mM GTPγS, 12 μM tubulin, and 45 μM Mal3_143_ at 37°C for 5 min before plunge freezing (see Figure S1A). For Mal3_143_-GDP microtubule complexes, microtubules were grown from quantum dot-labeled GMPCPP seeds in the presence of 2 mM GTP, 10 μM tubulin, and 55 μM Mal3_143_ at 37°C for 1 min before plunge freezing.

### Cryo-EM Data Collection and Image Processing

Low-dose images of Mal3_143_-GTPγS-microtubule complexes were collected on an electron microscope (Tecnai F20 FEG; FEI Company) operating at 200 kV, 68,000× magnification, and 0.7–3.6 μm defocus. Micrographs were recorded on a 4k × 4k CCD camera (Gatan) with a sampling of 2.2 Å/pixel. 162 CCD frames containing 312 microtubules were selected for 3D reconstruction, using a previously described custom single-particle procedure (Fourniol et al., 2010; Sindelar and Downing, 2010). The procedure included 305 microtubules (98%) in the final B lattice 13 protofilament microtubule reconstruction. To avoid model bias, the initial reference 3D model was a low-resolution microtubule decorated with a kinesin-1 motor domain. After pruning the data to achieve an isotropic angular distribution (Figure S1D), 244 microtubules (129,000 tubulin dimers) were used for the final reconstruction. Reconstructions from independent half-datasets confirmed the lateral contacts described in Figures 6 and S5. Low-dose images of Mal3_143_-GDP-microtubule complexes were collected on an electron microscope (Tecnai F30 FEG; FEI Company) operating at 200 kV, using settings similar to those above. The procedure included 344 microtubules (83%) in the B lattice 13 protofilament microtubule reconstruction. The lower percentage of inclusion compared with the GTPγS dataset (83% versus 98%) probably derives from noisier images due to the lower affinity of Mal3 for the GDP lattice.

### Pseudoatomic Model Building

UCSF Chimera (Pettersen et al., 2004) was used for visualization of 3D models and rigid-body fitting of atomic structures in the cryo-EM volume, using 1JFF.pdb (Löwe et al., 2001) for β-tubulin, a hybrid structure for α-tubulin as described previously (2XRP.pdb; Fourniol et al., 2010), and a homology model of the Mal3 CH domain generated using MODELER (Sali and Blundell, 1993) based on the structure of its ortholog Bim1 (2QJX.pdb; Slep and Vale, 2007). The multisubunit fitting was refined with Flex-EM (Topf et al., 2008), considering each subunit as a rigid body with a flexible α_1_ loop H10-S9. The pseudoatomic model yielded a high cross-correlation score of 0.908, calculated between an 8 Å map simulated from coordinates and the cryo-EM map. Coordinates of the pseudoatomic model were deposited in the Protein Data Bank (PDB) (entry 4ABO).

### Fluorescence Microscopy

TIRF microscopy of WT Mal3-GFP and single-point mutants on dynamically growing Alexa 568-labeled microtubules in the presence of GTP was performed essentially as described (Maurer et al., 2011). Final concentrations were 22 μM tubulin containing 10% Alexa 568-labeled tubulin and 30 or 300 nM Mal3-GFP WT or mutants. The temperature was 30°C. Simultaneously acquired dual-color time-lapse movies were recorded at a frame rate of 0.5 frames per second (100 ms exposure time). The mean intensities measured for mutants and WT at the comet maximum at growing microtubule ends and on the microtubule lattice distant from the end were calculated as described (Maurer et al., 2011). For the analysis of the Mal3-GFP intensity as a function of time before catastrophe, final concentrations were 20 μM tubulin containing 10% Cy5-labeled tubulin and 60 nM Mal3-GFP, and the final MgCl_2_ concentration was raised to 10 mM. Data acquisition was started immediately after flow-chamber assembly. Time-lapse movies at 1 frame per second (150 ms exposure time) were recorded for a total length of 600 s. The microtubule-end position was determined from the Cy5-tubulin channel using a modified version of the Matlab tracking software FIESTA (Ruhnow et al., 2011). The Mal3-GFP comet intensities were obtained using a 2D fit to the Mal3-GFP fluorescence signal in an area of 1 μm × 0.4 μm around the comet signal. The Mal3-GFP intensity of the lattice was extracted from an area of the same size starting at a distance of 1.5 μm from the microtubule end. The backgrounds for both the Mal3-GFP and the Cy5-tubulin channel were determined from parameters of the 2D fits used to detect the Mal3-GFP comet intensity and the Cy5-tubulin end position. All intensity values were background corrected before further calculations. Subsequently, the normalized, final comet intensity was calculated by subtracting the Mal3-GFP lattice intensity value from the comet intensity and then dividing by the microtubule-end intensity from the Cy5-tubulin channel. Sixty-two catastrophe events from twelve individual experiments were analyzed. The individual tracks of the microtubule-end position and the corresponding comet intensities were aligned at the catastrophe time point and averaged. To determine the catastrophe time point, walking-average slopes over a time window of 5 s were calculated along each individual microtubule-end position track. When the slope reached a negative value of at least −250 nm/s, the central point of the window was assumed to be the catastrophe time point.

Extended Experimental Procedures **Cryo-EM Imaging for Protofilament Number Analysis**

GTPγS microtubules were grown from quantum dot-labeled GMPCPP seeds in the presence of 1 mM GTPγS and 20 μM tubulin at 37°C for 30 min before plunge freezing. Low-dose images were collected on an electron microscope (Tecnai F30 FEG; FEI Company) operating at 300 kV, 59,000× magnification, and 3–5 μm defocus. The protofilament number and start of 148 GTPγS-MTs and 146 Mal3_143_-GTPγS-MTs were determined based on visual inspection of their moiré patterns (Chrétien and Fuller, 2000).

## Accession Numbers

Reconstructions were deposited in the Electron Microscopy Data Bank (EMDB) under the following accession numbers: 2004 (GTPγS asymmetric reconstruction), 2005 (GTPγS 8.6 Å reconstruction), and 2006 (GDP asymmetric reconstruction).

## Supplemental Information

Movie S1. Related to Figures 2, 3, and 6 8.6 Å reconstruction of the Mal3_143_-GTPγS microtubule interface docked with atomic coordinates of tubulin and Mal3 CH domain. The movie illustrates the central role of the β_3_-tubulin helix H3 (magenta). At the N terminus of helix H3, loop T3 (magenta) senses the γS group of the exchangeable nucleotide. At its C terminus, β_3_-tubulin helix H3 contacts the neighboring β_4_-tubulin (asterisk). The final frame shows the Mal3-H3 contacts in the pseudoatomic model of the Mal3 CH-microtubule interface (side chains of residues <5 Å apart displayed as ball and stick), viewed from the lumen outwards. Rendering parameters as in Figure 2.

Movie S2. Related to Figure 5 Time-lapse TIRF microscopy movies of WT Mal3-GFP and three Mal3 mutants on microtubules grown in GTP. Imaging conditions are identical to Figure 5.

Document S1. Article plus Supplemental Information

## References

[bib1] Akhmanova A., Steinmetz M.O. (2010). Microtubule +TIPs at a glance. J. Cell Sci..

[bib2] Bieling P., Laan L., Schek H., Munteanu E.L., Sandblad L., Dogterom M., Brunner D., Surrey T. (2007). Reconstitution of a microtubule plus-end tracking system in vitro. Nature.

[bib3] Bieling P., Kandels-Lewis S., Telley I.A., van Dijk J., Janke C., Surrey T. (2008). CLIP-170 tracks growing microtubule ends by dynamically recognizing composite EB1/tubulin-binding sites. J. Cell Biol..

[bib4] Browning H., Hackney D.D., Nurse P. (2003). Targeted movement of cell end factors in fission yeast. Nat. Cell Biol..

[bib5] Busch K.E., Brunner D. (2004). The microtubule plus end-tracking proteins mal3p and tip1p cooperate for cell-end targeting of interphase microtubules. Curr. Biol..

[bib6] Caplow M., Shanks J. (1996). Evidence that a single monolayer tubulin-GTP cap is both necessary and sufficient to stabilize microtubules. Mol. Biol. Cell.

[bib7] Carlier M.F., Hill T.L., Chen Y. (1984). Interference of GTP hydrolysis in the mechanism of microtubule assembly: an experimental study. Proc. Natl. Acad. Sci. USA.

[bib8] Chrétien D., Fuller S.D., Karsenti E. (1995). Structure of growing microtubule ends: two-dimensional sheets close into tubes at variable rates. J. Cell Biol..

[bib9] des Georges A., Katsuki M., Drummond D.R., Osei M., Cross R.A., Amos L.A. (2008). Mal3, the Schizosaccharomyces pombe homolog of EB1, changes the microtubule lattice. Nat. Struct. Mol. Biol..

[bib10] Desai A., Mitchison T.J. (1997). Microtubule polymerization dynamics. Annu. Rev. Cell Dev. Biol..

[bib11] Dimitrov A., Quesnoit M., Moutel S., Cantaloube I., Poüs C., Perez F. (2008). Detection of GTP-tubulin conformation in vivo reveals a role for GTP remnants in microtubule rescues. Science.

[bib12] Dixit R., Barnett B., Lazarus J.E., Tokito M., Goldman Y.E., Holzbaur E.L. (2009). Microtubule plus-end tracking by CLIP-170 requires EB1. Proc. Natl. Acad. Sci. USA.

[bib13] Drechsel D.N., Kirschner M.W. (1994). The minimum GTP cap required to stabilize microtubules. Curr. Biol..

[bib14] Fourniol F.J., Sindelar C.V., Amigues B., Clare D.K., Thomas G., Perderiset M., Francis F., Houdusse A., Moores C.A. (2010). Template-free 13-protofilament microtubule-MAP assembly visualized at 8 A resolution. J. Cell Biol..

[bib15] Galjart N. (2010). Plus-end-tracking proteins and their interactions at microtubule ends. Curr. Biol..

[bib16] Gardner M.K., Charlebois B.D., Jánosi I.M., Howard J., Hunt A.J., Odde D.J. (2011). Rapid microtubule self-assembly kinetics. Cell.

[bib17] Hayashi I., Ikura M. (2003). Crystal structure of the amino-terminal microtubule-binding domain of end-binding protein 1 (EB1). J. Biol. Chem..

[bib18] Hayashi I., Wilde A., Mal T.K., Ikura M. (2005). Structural basis for the activation of microtubule assembly by the EB1 and p150Glued complex. Mol. Cell.

[bib19] Helenius J., Brouhard G., Kalaidzidis Y., Diez S., Howard J. (2006). The depolymerizing kinesin MCAK uses lattice diffusion to rapidly target microtubule ends. Nature.

[bib20] Honnappa S., Okhrimenko O., Jaussi R., Jawhari H., Jelesarov I., Winkler F.K., Steinmetz M.O. (2006). Key interaction modes of dynamic +TIP networks. Mol. Cell.

[bib21] Honnappa S., Gouveia S.M., Weisbrich A., Damberger F.F., Bhavesh N.S., Jawhari H., Grigoriev I., van Rijssel F.J., Buey R.M., Lawera A. (2009). An EB1-binding motif acts as a microtubule tip localization signal. Cell.

[bib22] Hyman A.A., Salser S., Drechsel D.N., Unwin N., Mitchison T.J. (1992). Role of GTP hydrolysis in microtubule dynamics: information from a slowly hydrolyzable analogue, GMPCPP. Mol. Biol. Cell.

[bib23] Iimori M., Ozaki K., Chikashige Y., Habu T., Hiraoka Y., Maki T., Hayashi I., Obuse C., Matsumoto T. (2012). A mutation of the fission yeast EB1 overcomes negative regulation by phosphorylation and stabilizes microtubules. Exp. Cell Res..

[bib24] Katsuki M., Drummond D.R., Osei M., Cross R.A. (2009). Mal3 masks catastrophe events in Schizosaccharomyces pombe microtubules by inhibiting shrinkage and promoting rescue. J. Biol. Chem..

[bib25] Komarova Y., De Groot C.O., Grigoriev I., Gouveia S.M., Munteanu E.L., Schober J.M., Honnappa S., Buey R.M., Hoogenraad C.C., Dogterom M. (2009). Mammalian end binding proteins control persistent microtubule growth. J. Cell Biol..

[bib26] Komarova Y.A., Akhmanova A.S., Kojima S., Galjart N., Borisy G.G. (2002). Cytoplasmic linker proteins promote microtubule rescue in vivo. J. Cell Biol..

[bib27] Kueh H.Y., Mitchison T.J. (2009). Structural plasticity in actin and tubulin polymer dynamics. Science.

[bib28] Kukulski W., Schorb M., Welsch S., Picco A., Kaksonen M., Briggs J.A. (2011). Correlated fluorescence and 3D electron microscopy with high sensitivity and spatial precision. J. Cell Biol..

[bib29] Li H., DeRosier D.J., Nicholson W.V., Nogales E., Downing K.H. (2002). Microtubule structure at 8 A resolution. Structure.

[bib30] Löwe J., Li H., Downing K.H., Nogales E. (2001). Refined structure of alpha beta-tubulin at 3.5 A resolution. J. Mol. Biol..

[bib31] Mandelkow E.M., Mandelkow E., Milligan R.A. (1991). Microtubule dynamics and microtubule caps: a time-resolved cryo-electron microscopy study. J. Cell Biol..

[bib32] Maurer S.P., Bieling P., Cope J., Hoenger A., Surrey T. (2011). GTPgammaS microtubules mimic the growing microtubule end structure recognized by end-binding proteins (EBs). Proc. Natl. Acad. Sci. USA.

[bib33] McIntosh J.R., Morphew M.K., Grissom P.M., Gilbert S.P., Hoenger A. (2009). Lattice structure of cytoplasmic microtubules in a cultured Mammalian cell. J. Mol. Biol..

[bib34] Meurer-Grob P., Kasparian J., Wade R.H. (2001). Microtubule structure at improved resolution. Biochemistry.

[bib35] Mitchison T., Kirschner M. (1984). Dynamic instability of microtubule growth. Nature.

[bib36] Mizuno N., Toba S., Edamatsu M., Watai-Nishii J., Hirokawa N., Toyoshima Y.Y., Kikkawa M. (2004). Dynein and kinesin share an overlapping microtubule-binding site. EMBO J..

[bib37] Montenegro Gouveia S., Leslie K., Kapitein L.C., Buey R.M., Grigoriev I., Wagenbach M., Smal I., Meijering E., Hoogenraad C.C., Wordeman L. (2010). In vitro reconstitution of the functional interplay between MCAK and EB3 at microtubule plus ends. Curr. Biol..

[bib38] Nawrotek A., Knossow M., Gigant B. (2011). The determinants that govern microtubule assembly from the atomic structure of GTP-tubulin. J. Mol. Biol..

[bib39] Nogales E., Whittaker M., Milligan R.A., Downing K.H. (1999). High-resolution model of the microtubule. Cell.

[bib40] Pettersen E.F., Goddard T.D., Huang C.C., Couch G.S., Greenblatt D.M., Meng E.C., Ferrin T.E. (2004). UCSF Chimera—a visualization system for exploratory research and analysis. J. Comput. Chem..

[bib41] Ravelli R.B., Gigant B., Curmi P.A., Jourdain I., Lachkar S., Sobel A., Knossow M. (2004). Insight into tubulin regulation from a complex with colchicine and a stathmin-like domain. Nature.

[bib42] Reijo R.A., Cooper E.M., Beagle G.J., Huffaker T.C. (1994). Systematic mutational analysis of the yeast beta-tubulin gene. Mol. Biol. Cell.

[bib43] Rice L.M., Montabana E.A., Agard D.A. (2008). The lattice as allosteric effector: structural studies of alphabeta- and gamma-tubulin clarify the role of GTP in microtubule assembly. Proc. Natl. Acad. Sci. USA.

[bib44] Richards K.L., Anders K.R., Nogales E., Schwartz K., Downing K.H., Botstein D. (2000). Structure-function relationships in yeast tubulins. Mol. Biol. Cell.

[bib45] Ruhnow F., Zwicker D., Diez S. (2011). Tracking single particles and elongated filaments with nanometer precision. Biophys. J..

[bib46] Sali A., Blundell T.L. (1993). Comparative protein modelling by satisfaction of spatial restraints. J. Mol. Biol..

[bib47] Sandblad L., Busch K.E., Tittmann P., Gross H., Brunner D., Hoenger A. (2006). The Schizosaccharomyces pombe EB1 homolog Mal3p binds and stabilizes the microtubule lattice seam. Cell.

[bib48] Schek H.T., Gardner M.K., Cheng J., Odde D.J., Hunt A.J. (2007). Microtubule assembly dynamics at the nanoscale. Curr. Biol..

[bib49] Sindelar C.V., Downing K.H. (2010). An atomic-level mechanism for activation of the kinesin molecular motors. Proc. Natl. Acad. Sci. USA.

[bib50] Slep K.C., Vale R.D. (2007). Structural basis of microtubule plus end tracking by XMAP215, CLIP-170, and EB1. Mol. Cell.

[bib51] Sui H., Downing K.H. (2010). Structural basis of interprotofilament interaction and lateral deformation of microtubules. Structure.

[bib52] Tirnauer J.S., Grego S., Salmon E.D., Mitchison T.J. (2002). EB1-microtubule interactions in Xenopus egg extracts: role of EB1 in microtubule stabilization and mechanisms of targeting to microtubules. Mol. Biol. Cell.

[bib53] Topf M., Lasker K., Webb B., Wolfson H., Chiu W., Sali A. (2008). Protein structure fitting and refinement guided by cryo-EM density. Structure.

[bib54] Vetter I.R., Wittinghofer A. (2001). The guanine nucleotide-binding switch in three dimensions. Science.

[bib55] Vitre B., Coquelle F.M., Heichette C., Garnier C., Chrétien D., Arnal I. (2008). EB1 regulates microtubule dynamics and tubulin sheet closure in vitro. Nat. Cell Biol..

[bib56] Wang H.W., Nogales E. (2005). Nucleotide-dependent bending flexibility of tubulin regulates microtubule assembly. Nature.

[bib57] Zanic M., Stear J.H., Hyman A.A., Howard J. (2009). EB1 recognizes the nucleotide state of tubulin in the microtubule lattice. PLoS ONE.

[bib58] Zimniak T., Stengl K., Mechtler K., Westermann S. (2009). Phosphoregulation of the budding yeast EB1 homologue Bim1p by Aurora/Ipl1p. J. Cell Biol..

