# Supplementary figures and images for: EBs Recognize a Nucleotide-Dependent Structural Cap at Growing Microtubule Ends
Source: Cell. 2012 Apr 13;149(2-2):371–82. doi: 10.1016/j.cell.2012.02.049 (PMC3368265; doi:10.1016/j.cell.2012.02.049)

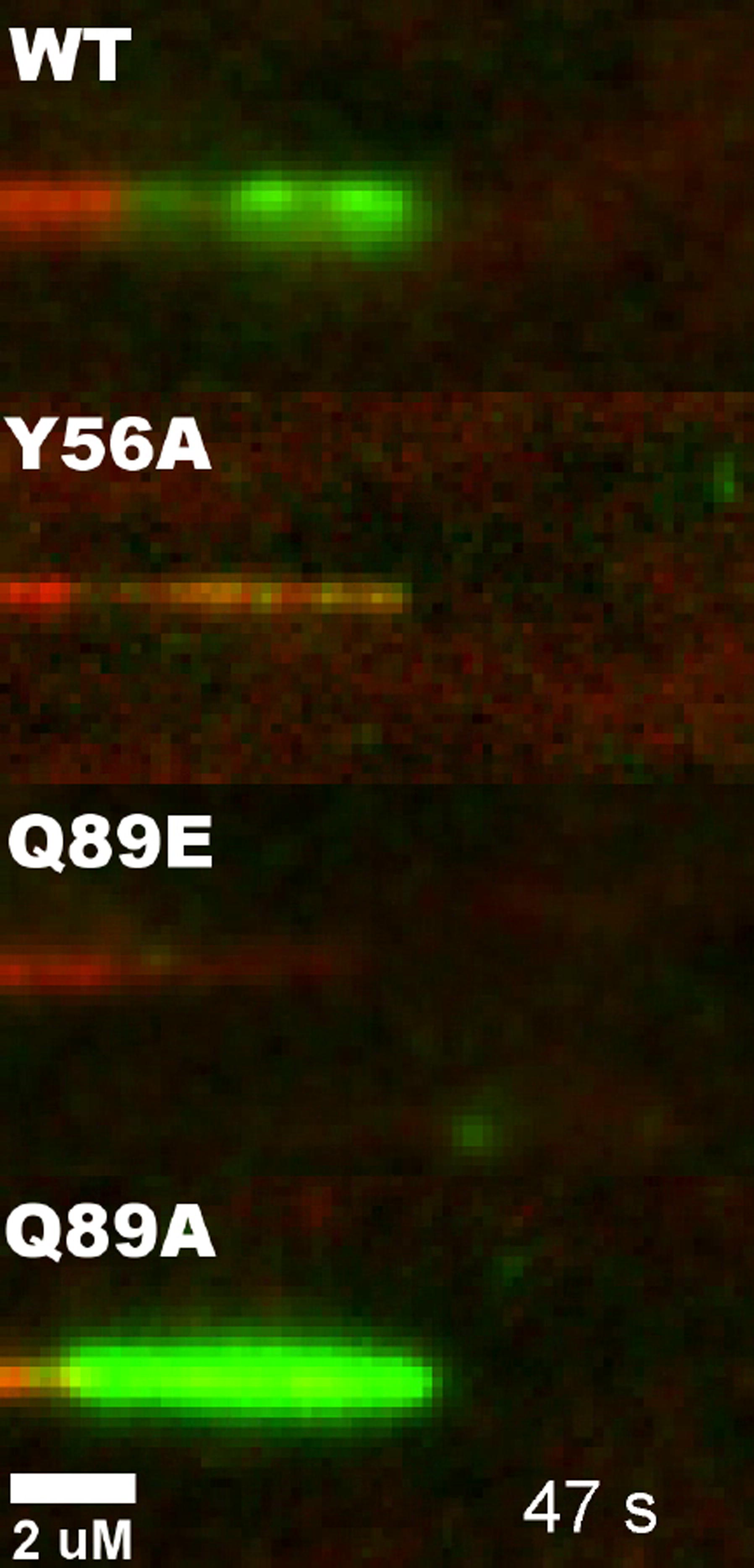

Supplement: Movie S2. Related to Figure 5 — Time-lapse TIRF microscopy movies of WT Mal3-GFP and three Mal3 mutants on microtubules grown in GTP. Imaging conditions are identical to Figure 5. [file mmc2.jpg]
